# Potential Treat-to-Target Approach for Methamphetamine Use Disorder: A Pilot Study of Adenosine 2A Receptor Antagonist With Positron Emission Tomography

**DOI:** 10.3389/fphar.2022.820447

**Published:** 2022-05-11

**Authors:** Kyoji Okita, Toshihiko Matsumoto, Daisuke Funada, Maki Murakami, Koichi Kato, Yoko Shigemoto, Noriko Sato, Hiroshi Matsuda

**Affiliations:** ^1^ Department of Psychiatry, Center Hospital, National Center of Neurology and Psychiatry, Tokyo, Japan; ^2^ Department of Drug Dependence Research, National Institute of Mental Health, National Center of Neurology and Psychiatry, Tokyo, Japan; ^3^ Integrative Brain Imaging Center, National Center of Neurology and Psychiatry, Tokyo, Japan; ^4^ Department of Radiology, Center Hospital, National Center of Neurology and Psychiatry, Tokyo, Japan; ^5^ Drug Discovery and Cyclotron Research Center, Southern TOHOKU Research Institute for Neuroscience, Fukushima, Japan; ^6^ Department of Biofunctional Imaging, Fukushima Medical University, Fukushima, Japan

**Keywords:** methamphetamine, addiction, dopamine D2 receptors, adenosine 2A receptor, neuroinflammation, positron emission tomography

## Abstract

**Introduction:** The misuse of stimulant drugs such as methamphetamine is a global public health issue. One important neurochemical mechanism of methamphetamine use disorder may be altered dopaminergic neurotransmission. For instance, previous studies using positron emission tomography (PET) have consistently shown that striatal dopamine D2-type receptor availability (quantified as binding potential; BP_ND_) is lower in methamphetamine use disorder. Further, methamphetamine use is known to induce chronic neuroinflammation through multiple physiological pathways. Upregulation of D2-type receptor and/or attenuation of neuroinflammation may therefore provide a therapeutic effect for this disorder. *In vitro* studies have shown that blockage of adenosine 2A (A2A) receptors may prevent D2-receptor downregulation and neuroinflammation-related brain damage. However, no study has examined this hypothesis yet.

**Methods and Analysis:** Using a within-subject design, this trial will assess the effect of the selective A2A receptor antagonist, istradefylline, primarily on D2-type BP_ND_ in the striatum, and secondarily on neuroinflammation in the whole brain in individuals with methamphetamine use disorder. The research hypotheses are that istradefylline will increase striatal D2-type BP_ND_ and attenuate neuroinflammation. Twenty participants with methamphetamine use disorder, aged 20–65, will be recruited to undergo [^11^C]raclopride PET (for every participant) and [^11^C]DAA1106 PET (if applicable) once before and once after administration of 40 mg/day istradefylline for 2 weeks. Neuropsychological measurements will be performed on the same days of the PET scans.

## Introduction

Misuse of stimulant drugs such as methamphetamine is a worldwide public health issue. Methamphetamine use is deemed to associate with not only physical harms including HIV transmission (Shoptaw and Reback, 2007), cardiovascular disease (Darke et al., 2017; Kevil et al., 2019), cerebrovascular disease (Lappin et al., 2017) but also psychiatric harms including psychosis, depression, anxiety and suicide In 2019, it is estimated that as much as 0.5% of global population aged between 15 and 64 had used the amphetamine-type stimulants [United Nations Office on Drugs and Crime (UNODC), 2021]. From 2010 to 2018, the prevalence of methamphetamine use within the general population in the United States over the past year has increased by 195% (Paulus and Stewart, 2020), and the number of overdose deaths attributed to psychostimulant use has tripled over the previous 5 years in 2019 (Han et al., 2021). Despite the negative effects of such methamphetamine use on society, current treatments are inadequate, and no pharmacological treatments have been approved for any stimulant use disorders. Understanding the biological basis of methamphetamine use may aid the development of novel therapies for methamphetamine use disorder.

Dopaminergic neurotransmission via actions at D2-type receptors has been suggested to play an important role in addictive disorders (Solinas et al., 2019). For instance, lower striatal dopamine D2-type receptor availability (quantified as binding potential; BP_ND_) has been consistently observed in individuals with methamphetamine use disorder *vs*. healthy-control subjects in studies using positron emission tomography (PET) [see reviews by (Volkow et al., 2009; Ashok et al., 2017; London, 2020)]. Furthermore, in methamphetamine users, striatal D2-type BP_ND_ is negatively correlated with higher scores on the Barratt Impulsiveness Scale (Lee et al., 2009), a greater choosing of methamphetamine-related images over pleasant images compared to healthy-control subjects in a computer-based picture choice task (Moeller et al., 2018), and even possibly with treatment outcomes (i.e., relapse to methamphetamine use) (Wang et al., 2012).

As such, enhancing neurotransmission through striatal D2-type receptors may create a therapeutic effect for methamphetamine use disorder. D2-type receptor agonists have failed to show a therapeutic effect though (Verrico et al., 2013; Blum et al., 2014), and no pharmacological treatment has been approved for the disorder to date. Importantly, these evidences may suggest that agonistic effects on D2-type receptors are not enough while recovering the function of these receptors (i.e., upregulation of D2-type receptor density) is needed to provide a therapeutic effect.

Adenosine 2A (A2A) receptors, as well as dopamine D2-type receptors, are G protein-coupled receptors (GPCR), and they are expressed on the dendritic spines of striatopallidal GABAergic neurons forming A2A-D2 receptor heteromers in the whole striatum with a higher density than any other brain regions. They functionally interact with each other in the heteromer structure (Ferre et al., 2016). Treatment with selective A2A receptor antagonists attenuates D2 receptor internalization from the membrane into the cell body of cultivated rat striatal neurons (Huang et al., 2013). Thus, A2A receptor antagonism could block internalization of D2 receptors in the human brain and may induce upregulation of these receptors in the individuals with methamphetamine use disorder.

Importantly, methamphetamine use is known to induce chronic neuroinflammation through multiple physiological pathways [see review by (Kim et al., 2020)]. Neuroinflammation is considered to activate microglia, which can be assessed using PET scans with tracers that target the translocator protein 18 kDa (TSPO) *in vivo* (Meyer et al., 2020). Indeed, compared to healthy non-drug-using controls, methamphetamine users who had refrained from methamphetamine use for 6 months–4 years exhibited significantly higher microglial activation in a PET study that used a first generation TSPO tracer [^11^C]PK11195 (Sekine et al., 2008). Further, compared to healthy controls, methamphetamine users who were abstinent for only 4–7 days also exhibited greater standardized uptake value (SUV) measured with [^11^C]DAA1106, a second generation TSPO tracer with higher affinity and specificity than [^11^C]PK11195, although this group differences did not reach significance likely due in part to the small sample size (London et al., 2020).

Previous basic studies have shown evidence that blockade of A2A receptors can have anti-inflammatory and neuroprotective effects in the rodent brain (Pierri et al., 2005; Gołembiowska et al., 2013; Ogawa et al., 2018; Zhou et al., 2019). However, no study has yet attempted to evaluate the effects of A2A receptor antagonism on neuroinflammation in the human brain.

Istradefylline is a selective A2A-receptor antagonist, and it received the drug approval as an add-on treatment to levodopa and/or carbidopa in adult patients with Parkinson’s disease in 2013 by Ministry of Health, Labour and Welfare in Japan (Dungo and Deeks, 2013), and in 2019 by the U.S. Food and Drug Administration (Chen and Cunha, 2020). Istradefylline is a structural analog of caffeine and is characterized by long-term safety and high tolerability (Kondo and Mizuno, 2015). When administered in combination with levodopa for patients with Parkinson’s disease in a phase I clinical trial, dyskinesia was the most frequently reported side effect (16.9% of participants), although when istradefylline was administered alone to healthy individuals there were no clinically significant adverse events reported (Kirin, 2013). These features would fit for purpose of treating methamphetamine use disorder that requires long term commitment, although the risk of unexpected effect should not be ignored because it is an off-label use and, to our knowledge, this study would be the first trial of istradefylline for methamphetamine use disorder.

The main aim of this study is to evaluate if medium-term treatment with istradefylline can induce D2-type receptor upregulation in striatum in individuals with methamphetamine use disorder (Aim 1). It is hypothesized that this pharmacological intervention will increase D2-type BP_ND_ as measured with [^11^C]raclopride scan; such increases will be indicated by within-subject differences (i.e., pre- *vs.* post- intervention). The secondary aim is to evaluate the effects of this pharmacological intervention on neuroinflammation (Aim 2). As such, the secondary hypothesis is that whole brain SUV (as quantified by the [^11^C]DAA1106 scan) will decrease after 2-week administration of istradefylline. Lastly, further aims are to evaluate the extent to which any effects on cognitive performance and/or risk of recurrent methamphetamine use are associated with changes in D2-type receptor (Aim 3) or neuroinflammation (Aim 4). The hypotheses pertaining to these aims are that 2-week administration of istradefylline would both improve neurocognitive measures and attenuate the risk of recurrent methamphetamine use as indicated by within-subject pre- *vs*. post-administration difference, and that any effects of the pharmacological intervention on D2-type BP_ND_ measured with [^11^C]raclopride and/or SUV of [^11^C]DAA1106 will be associated with such effects on cognitive performance and risk of recurrent use.

## Methods and Analysis

### Research Design

**FIGURE 1:**
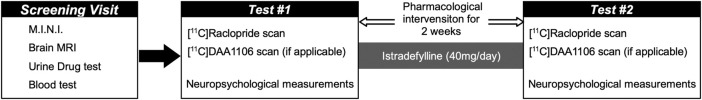
Schematic diagram of the study.

This study will employ a within-subject design and aims to determine the potential effect of istradefylline, a selective A2A receptor antagonist, firstly on striatal D2-type BP_ND_, and secondarily on neuroinflammation in the whole brain. Participants will receive 40 mg/day of istradefylline for 2 weeks and will undergo two PET scans; one before and one after administration of istradefylline for 2 weeks. All participants will undergo [^11^C]raclopride PET scans and neurocognitive tests (Figure 1). Importantly, if a participant is strongly averse to undergoing a [^11^C]DAA1106 scan, due to any reason (i.e., if they are anxious about radiation exposure), or if they meet the additional exclusion criterion for a neuroinflammation PET scan (see below for more details), they will not undergo the PET scans.

### Participants

Twenty participants with methamphetamine use disorder will be recruited for this study. Inclusion criteria are: being between 20 and 65 years old, being able to comprehend Japanese, and meeting criteria for methamphetamine use disorder, according to the DSM-5. Exclusion criteria include the following: lifetime history or current neurological disorder; past history of psychiatric disorder which precedes initiation of methamphetamine use [confirmed using the Mini International Neuropsychiatric Interview (M.I.N.I.)]; (an) intracranial lesion(s) as observed from the structural magnetic resonance image (MRI); impairment of liver and/or renal function; HIV seropositive; positive urine-sample test for illegal drugs other than methamphetamine at the screening visit; past history of suicidal ideation or attempt; claustrophobia or aichmophobia; metals inside the body incompatible with MRI; use of psychotropic drugs which could interfere D2-type BP_ND_ measurement such as antipsychotics; use of drugs that interact with CYP3A4, a major metabolizing enzyme of istradefylline. Also, individuals will be required to abstain from methamphetamine use during participation in the study; this abstinence will be confirmed with urine sample test on each day of PET scans (Supplementary Table S1). Additionally, for those who will undergo the neuroinflammation scan with [^11^C]DAA1106, the use of anti-inflammatory drugs such as NSAIDs, or current tobacco smoking, which have been proven to influence neuroinflammation (Brody et al., 2017; Brody et al., 2018) will be an exclusion criterion.

### Research Ethics and Recruitment

This study will take place at the National Center of Neurology and Psychiatry (NCNP) in Kodaira, Tokyo, Japan. The study procedure has been reviewed and approved by the Certified Review Boards (CRB) of the NCNP. Study participants will be recruited at an outpatient clinic run by Drug Dependence Center at the NCNP. Potential participants will contact the research team by email or phone. After brief screening, those who meet inclusion/exclusion criteria will then be invited to come into the office for an in-person screening. At the initial visit, participants will be given a full description of the study procedure and will be asked to sign the CRB-approved informed consent forms with the PI. Comprehensive baseline assessments including M.I.N.I., urine drug test, blood test and MRI scan (see below for details) then will be performed for rigorous screening. Medical eligibility for the study will be determined by the PI *via* examination of all data acquired at the screening visit and clinical record.

### Patient and Public Involvement

Participants will not be involved in the recruitment, conduct of the study and decision of which data and results to report. While, members of the research team may listen to input from participants during the process of deciding what data to report, the final decision as to which findings to report will be made only by the scientists.

### Study Medication

Participants will receive 40 mg/day of istradefylline for 2 weeks. This dose was selected because it is the maximum dose approved in clinical practice, and this dose has been shown to be tolerable for healthy individuals in a phase I clinical trial of istradefylline that used a similar administration protocol as our own (i.e., 2-week administration of 20, 40, or 80 mg) (Kyowa Kirin, 2013). Istradefylline (brand name Nourianz^®^; Kyowa Kirin, Inc., Tokyo, Japan) will be provided by a study pharmacist at the Department of Pharmacology of the NCNP hospital. Participants will receive an email notification from the investigator team every morning to check adherence and potential side effect.

### Neuroimaging

#### Magnetic Resonance Image Scans

Structural MRI scans of the brain for co-registration with PET images and definition of volumes-of-interest (VOIs) will be acquired using Achieva 3.0T TX (Koninklijke Philips N.V., Amsterdam, Netherland). A T1-weighted scan will be acquired using a whole-brain magnetization-prepared rapid acquisition with gradient echo (MPRAGE) (TR = 7.223 ms, TE = 3.468 ms, flip angle = 10, field of view = 384 × 384 × 300, 300 slices, thickness = 0.6 mm).

#### Positron Emission Tomography Scans

Biograph TruePoint 6 PET/CT (Siemens Healthineers, Erlangen, Germany), which has a resolution full-width at half-maximum (FWHM) of 4.1 mm, and an axial field of view of 162 mm in the 3D mode, will be used for PET data acquisition. Low-dose CT scan for attenuation correction firstly will be performed, then emission data will be collected with a dynamic mode for 60 min after a bolus injection of 10 mCi (±10%; range of 9–11 mCi) [^11^C]raclopride for measurement of dopamine D2-type receptor availability (Kohler et al., 1985), which has a specific activity of >1 Ci/μmol. For neuroinflammation measurement, emission data collection will start 20 min after injection of 10 mCi (±10%; range of 9–11 mCi, specific activity of >1 Ci/μmol) [^11^C]DAA1106, a second-generation radiotracer for labeling translocator protein (TSPO) with high affinity (Maeda et al., 2004; Venneti et al., 2008).

### Data Processing

#### Magnetic Resonance Image Data

VOIs will be derived from individual MPRAGE images. For [^11^C]raclopride scan, striatum VOI, including caudate and putamen, will be created using FSL FIRST (Patenaude et al., 2011). As the reference region (Hall et al., 1994), cerebellum VOI, including the hemispheres but not the vermis, will be manually created in standard space (MNI152 template) and transformed into native space with FSL FNIRT. For [^11^C]DAA1106 scan, whole brain VOI will be created using FSL BET.

#### Positron Emission Tomography Data

The PET data reconstruction will use the manufacturer’s 3-dimention ordered subset expectation maximization (3D-OSEM) algorithm (Lee et al., 2014). The 60-min [^11^C]raclopride emission data will be reconstructed into twelve 20-s frames, sixteen 60-s frames, and ten 240-s frames (Kubota et al., 2017), and the 20-min [^11^C]DAA1106 data will be reconstructed into twelve 60-s frames. Motion correction between the frames will be performed with FSL MCFLIRT (FMRIB Centre, Dept. Clinical Neurology, University of Oxford) (Jenkinson et al., 2002). The images will then be co-registered to the MPRAGE image using a 6-parameter, rigid-body spatial transformation (FSL FLIRT).

#### BP_ND_ for [^11^C]raclopride Scan

Time-activity data within striatal and cerebellum VOIs will be extracted from the reconstructed frames of [^11^C]raclopride scan and imported into PMOD Kinetic Modeling (PKIN) tool (PMOD Technologies Ltd., Zurich, Switzerland). The simplified reference tissue model (SRTM) (Lammertsma and Hume, 1996) will be used to calculate BP_ND_ with time–activity curves from VOIs as follows: C_T_(t) = R1C_R_(t) + [k2 – R1k2/(1 + BP_ND_)] C_R_(t) * exp [−k2t/(1 + BP_ND_)], where C_T_(t) is the total radioactivity concentration in the striatum VOI measured by PET, R1 is the ratio of K1 (the influx rate constant for the striatum) to K1’ (the influx rate constant for the cerebellum), C_R_(t) is the radioactivity concentration in the reference region (cerebellum), and * denotes the convolution integral. The parameters R1, k2, and BP_ND_ in this model are estimated by a nonlinear curve-fitting procedure.

#### Standardized Uptake Values for [^11^C]DAA1106 Scan

For measurements of [^11^C]DAA1106 binding to TSPO in whole brain, standardized uptake values (SUV) will be used as the primary outcome measure because it avoids invasive arterial blood sampling. It will be calculated using the standard definition of SUV = mean tissue activity concentration (Bq/mL)/[(injected dose (Bq)/body weight (g)]. Mean activity in whole brain from 20 to 40 min post-injection was used, based on time-activity curves from our previous study demonstrating stable activity during this time period (Brody et al., 2017; Brody et al., 2018; London et al., 2020).

### Neuropsychological Measurements

The following measurements will be administered on each day of [^11^C]raclopride scan.

• Stimulant Relapse Risk Scale (SRRS): This 30-item self-report questionnaire, which was developed to measure stimulant relapse risk in Japanese language, will be used. The SRRS is composed of five subscales: anxiety and intention to use drugs, emotionality problems, compulsivity for drug use, positive expectancies, lack of control over drug use and lack of negative expectancy for drug use (Ogai et al., 2007).
• Barratt Impulsiveness Scale (BIS): This 30-item self-report questionnaire assesses impulsive personality traits (Patton et al., 1995). The BIS has been shown to be sensitive to changes in impulsivity over time in individuals with methamphetamine use disorder (Ghahremani et al., 2013).
• Stop-Signal Task: Participants will be asked to press a left or right key in response to visual stimuli (left or right-direction arrow) presented on a laptop screen, whilst they will be instructed to withhold pressing the key if a tone (i.e., stop-signal) will be presented after a short delay after the visual stimuli. The primary dependent variable, stop-signal reaction time, indicates individual’s capability of response inhibition and it associates with impulsiveness.
• Monetary Choice Questionnaire (MCQ): Participants will be instructed to select between one of two hypothetical options in 27 questions to receive a certain amount of money immediately or a larger amount later. The discrepancies between the monetary amounts and duration of the delay are varied across questions. The primary dependent variable is the indifference point, or total k value, determined from the participant’s selections across the task (Kirby et al., 1999).
• Toronto Alexithymia Scale-20 (TAS-20): This self-administered questionnaire will be used to assess emotional self-awareness (Bagby et al., 1994). It a widely used questionnaire including for methamphetamine use disorder (Payer et al., 2011; Okita et al., 2016)Total score is interpreted as follows: 0–51: no alexithymia; 52–60: possible alexithymia; 61 and above: alexithymia (Taylor et al., 1999).
• Difficulties in Emotion Regulation Scale (DERS): This 36-item self-administered questionnaire will be used to assess emotion dysregulation (Gratz and Roemer, 2004). Total of the questionnaire ranges from 30 to 180 and higher scores represent more difficulties in emotion regulation.
• Beck Depression Inventory (BDI) (Beck et al., 1996) and Beck Anxiety Inventory (BAI) (Beck et al., 1988): The BDI and BAI are self-report inventories to verify depressive and anxiety symptoms respectively. They will be employed to verify exclusion criteria and monitor potential adverse events related to drug administration.

### Data Analytic Plan

#### Power Considerations

A sample size was selected based on a power analysis that was performed in advance. A previous study that showed D2-type receptor upregulation in humans after completion of an exercise intervention revealed a significant increase of striatal D2-type BP_ND_ with a Cohen’s dz = 0.97 (Robertson et al., 2016). Test-retest reliability of the [^11^C]raclopride PET scan has been shown good with a interclass correlation coefficient of 0.8 (Alakurtti et al., 2015). Given those numbers, a sample size of sixteen participants is deemed necessary to provide sufficient power (*β* > 0.95) to detect an effect using a critical 2-sided significance of *α* = 0.05. We set a sample size of N = 20 taking into account the potential participant drop-out rate of roughly 20%, which is common based on our previous studies (Moeller et al., 2018; Okita et al., 2018; London et al., 2020).

#### Statistical Analysis

We will use graphical and numerical summaries to screen the data for outliers and violations of model assumptions. For Aims 1 and 2, the hypotheses will be tested by evaluating the effects of time (pre- *vs*. post-drug administration) on striatal D2-type BP_ND_ and whole brain SUV (which will be the dependent variable) using paired samples *t*-tests. For Aims 3 and 4, the hypotheses will be tested using partial correlation analyses which will examine the associations between A) pre- *vs*. post-drug administration changes in BP_ND_ and SUV and B) changes in neurocognitive measures. These statistical analyses will all be conducted using SPSS IBM 25 (IBM, Armonk, NY, United States).

### Adverse Event Reporting

An annual summary of adverse events will be submitted to the Japan Registry of Clinical Trials (jRCT) and the CRB at NCNP. In the event that significant medical problems occur, or in the case that an investigator believes that any aspects of the study may be causing harm to participants’ health, the PI will promptly report all severe adverse events to the director of NCNP, the CRB of NCNP, the Pharmaceuticals and Medical Devices Agency, and the Ministry of Health, Labor and Welfare (where appropriate). Significant medical problems are defined as any fatal event, any immediately life-threatening event, any permanent or substantially disabling event, any event that requires or prolongs inpatient hospitalization, any congenital anomaly, or any unexpected adverse drug experiences that have not previously been observed.

### Data Monitoring

An independent review board at the Department of Clinical Epidemiology, Translational Medical Center at NCNP will perform regular monitoring over the course of the study. They will oversee the case report forms to verify that all study procedures are in compliance with approved study protocols, and will provide monitoring reports to the PI.

## Discussion

Substance use disorders are characterized by impaired dopaminergic neurotransmission. Notably, previous PET studies have repeatedly shown lower striatal D2-type BP_ND_ in individuals with substance use disorders *vs*. healthy control subjects [see reviews (Volkow et al., 2009; Ashok et al., 2017; London, 2020)]. Although the lower striatal D2-type BP_ND_ may reflect intrinsic biological aspect of individuals with substance use disorders, given that the phasic dopamine release in the human brain is induced by acute administration of most addictive substances (Nutt et al., 2015), and that agonism of GPCRs promotes desensitization of these receptors which possibly lead to their downregulation (Ferguson, 2001; Tabor et al., 2017), it seems plausible that chronic substance use could contribute to D2-receptor downregulation (Volkow et al., 2004). Further, *in vitro* studies using cultured cells derived from human neuroblastoma have revealed that coaggregation and co-internalization of A2A receptors and D2 receptors are induced by concurrent agonist stimulation of those receptors (Hillion et al., 2002; Bartlett et al., 2005). We therefore hypothesize that administration of A2A receptor antagonists for methamphetamine use disorder helps to recover from impaired dopaminergic neurotransmission through D2-receptor by producing D2 receptor upregulation.

In a human PET study using [^11^C]raclopride, which is the same ligand as we will use in this proposed study, a single administration of caffeine increased striatal D2-type BP_ND_ in healthy individuals (Volkow et al., 2015). However, it is unclear if this D2-type BP_ND_ change was a consequence of A2A receptor antagonist-induced D2 receptor internalization as caffeine acts as an antagonist of adenosine 2A receptor subtype as well as other subtypes of adenosine receptors (Ribeiro and Sebastiao, 2010). To eliminate this ambiguity, we are currently running a double-blind, randomized controlled trial assessing the effect of 2-week administration of istradefylline using the same PET scans as planed in this study on healthy individuals, that are not included in this study (Okita et al., 2021). Still, there is also a lack of evidence that A2A receptor antagonism increases D2-type BP_ND_ in addictive disorders, or whether such potential increases are associated with characteristics of addictive disorders such as magnitude of use and level of dependence. Thus, the main aim of this proposed study is to monitor changes in D2-type BP_ND_ in striatum due to istradefylline administration in individuals with methamphetamine use disorder.

As already mentioned, medications that have agonistic effect on dopamine D2-type receptors have failed to show a therapeutic effect (Verrico et al., 2013; Blum et al., 2014). It might be because those agonists are not able to mimic physiological dopaminergic function, in that they simply keep stimulating neurotransmission through D2-type receptors as long as they are in the central nervous system, regardless of the natural reaction of the dopaminergic system to reward. It has been suggested that the reward system of individuals with substance use disorders predominantly react to the substance addicted more than they react to natural rewards (Koob and Le Moal, 2005; Volkow et al., 2010; Hommer et al., 2011). This malfunctioning of the brain’s reward system is thought to be associated with impaired dopaminergic neurotransmission caused by chronic addictive substance use (Volkow et al., 2019). Thus, upregulation of dopamine D2-type receptor density may be beneficial by making the reward systems of methamphetamine-addicted individuals more reactive to other, non-drug-related stimuli, which may in turn provide a therapeutic effect for addictive disorders.

In addition, it has been suggested that the use of methamphetamine can produce neurotoxicity (Miyazaki et al., 2006; Shaerzadeh et al., 2018), which in turn can induce pathological brain changes and possibly cognitive deficits (London et al., 2015). Evidence from basic research has shown methamphetamine causes activation of microglia and astrocytes that produce inflammatory cytokines such as tumor necrosis factor and interleukin (Yamamoto and Raudensky, 2008). Further, higher neuroinflammation levels (represented by microglia activation) have been observed in individuals with methamphetamine use disorder (Sekine et al., 2008; London et al., 2020). Given the presumed important role of neuroinflammation in neuronal damage due to chronic methamphetamine use (Kim et al., 2020) and the suppressing effects of A2A receptor antagonist on neuroinflammation (Pierri et al., 2005; Ogawa et al., 2018; Zhou et al., 2019), it is worth evaluating potential changes in neuroinflammation beside dopamine D2-type receptors.

This study is not without limitations. The randomized placebo-control design would obviously be suitable to assess the medication’s effect. We are running another study of healthy individuals, that are not included in this proposed study, using a randomized double-blind placebo-controlled design (Okita et al., 2021). The purpose of the study in progress for healthy individuals is to verify a potential effect of istradefylline on D2-type receptors in human brain. However, the ultimate goal of a series of studies including the study in progress and this proposed study is to develop medication for methamphetamine use disorder. Because the randomized design requires a larger sample size than a study which employs a within-subject design, and because it is difficult to recruit many individuals with methamphetamine use disorder due to many such users failing to satisfy criterion of this D2-type receptor PET study (i.e., not taking antipsychotic medications, HIV negative and can keep abstinence for 2 weeks), we chose to perform a feasibility study due to limited human and budget resources. Despite this fundamental limitation, positive finding of D2-receptor upregulation from this study would have significance for the development of novel treatments for addictive disorders, particularly those that improve the functioning of dopamine D2-type receptors. Also, potential attenuation effect on neuroinflammation of istradefylline would have significance not only for addictive disorders but for neuropsychiatric disorders characterized by neuroinflammation.
